# Sphingolipid metabolites as potential circulating biomarkers for sarcopenia in men

**DOI:** 10.1002/jcsm.13582

**Published:** 2024-09-04

**Authors:** Je Hyun Seo, Jung‐Min Koh, Han Jin Cho, Hanjun Kim, Young‐Sun Lee, Su Jung Kim, Pil Whan Yoon, Won Kim, Sung Jin Bae, Hong‐Kyu Kim, Hyun Ju Yoo, Seung Hun Lee

**Affiliations:** ^1^ Veterans Health Service Medical Center Veterans Medical Research Institute Seoul South Korea; ^2^ Department of Internal Medicine, Division of Endocrinology and Metabolism, Asan Medical Center University of Ulsan College of Medicine Seoul South Korea; ^3^ Biomedical Research Center Asan Institute for Life Sciences, Asan Medical Center Seoul South Korea; ^4^ Department of Convergence Medicine, Asan Institute for Life Sciences, Asan Medical Center University of Ulsan College of Medicine Seoul South Korea; ^5^ Department of Orthopedic Surgery Seoul Now Hospital Anyang South Korea; ^6^ Department of Rehabilitation Medicine, Asan Medical Center University of Ulsan College of Medicine Seoul South Korea; ^7^ Health Screening and Promotion Center, Asan Medical Center University of Ulsan College of Medicine Seoul South Korea

**Keywords:** Aging, Biomarkers, Metabolomics, Sarcopenia, Sphingolipid

## Abstract

**Background:**

Sarcopenia is an age‐related progressive loss of muscle mass and function. Sarcopenia is a multifactorial disorder, including metabolic disturbance; therefore, metabolites may be used as circulating biomarkers for sarcopenia. We aimed to investigate potential biomarkers of sarcopenia using metabolomics.

**Methods:**

After non‐targeted metabolome profiling of plasma from mice of an aging mouse model of sarcopenia, sphingolipid metabolites and muscle cells from the animal model were evaluated using targeted metabolome profiling. The associations between sphingolipid metabolites identified from mouse and cell studies and sarcopenia status were assessed in men in an age‐matched discovery (72 cases and 72 controls) and validation (36 cases and 128 controls) cohort; women with sarcopenia (36 cases and 36 controls) were also included as a discovery cohort.

**Results:**

Both non‐targeted and targeted metabolome profiling in the experimental studies showed an association between sphingolipid metabolites, including ceramides (CERs) and sphingomyelins (SMs), and sarcopenia. Plasma SM (16:0), CER (24:1), and SM (24:1) levels in men with sarcopenia were significantly higher in the discovery cohort than in the controls (all *P* < 0.05). There were no significant differences in plasma sphingolipid levels for women with or without sarcopenia. In men in the discovery cohort, an area under the receiver‐operating characteristic curve (AUROC) of SM (16:0) for low muscle strength and low muscle mass was 0.600 (95% confidence interval [CI]: 0.501–0.699) and 0.647 (95% CI: 0.557–0.737). The AUROC (95% CI) of CER (24:1) and SM (24:1) for low muscle mass in men was 0.669 (95% CI: 0.581–0.757) and 0.670 (95% CI: 0.582–0.759), respectively. Using a regression equation combining CER (24:1) and SM (16:0) levels, a sphingolipid (SphL) score was calculated; an AUROC of the SphL score for sarcopenia was 0.712 (95% CI: 0.626–0.798). The addition of the SphL score to HGS significantly improved the AUC from 0.646 (95% CI: 0.575–0.717; HGS only) to 0.751 (95% CI: 0.671–0.831, *P* = 0.002; HGS + SphL) in the discovery cohort. The predictive ability of the SphL score for sarcopenia was confirmed in the validation cohort (AUROC = 0.695, 95% CI: 0.591–0.799).

**Conclusions:**

SM (16:0), reflecting low muscle strength, and CER (24:1) and SM (16:0), reflecting low muscle mass, are potential circulating biomarkers for sarcopenia in men. Further research on sphingolipid metabolites is required to confirm these results and provide additional insights into the metabolomic changes relevant to the pathogenesis and diagnosis of sarcopenia.

## Introduction

Sarcopenia is the progressive loss of muscle mass and function with age.^1^, ^2^, ^3^ Sarcopenia is associated with adverse outcomes, including falls, functional decline, frailty, and mortality.^1^, ^2^, ^3^ Therefore, sarcopenia has emerged as a major health concern, causing a huge economic burden.^4^ Currently, there are no specific drugs approved for treating sarcopenia, and the therapeutic effects of exercise and nutritional supplementation are limited after progression^3^; hence, detecting sarcopenia at an early stage is important for its management.

Diagnosing sarcopenia requires using various modalities to measure muscle mass, strength, and physical performance.^1^, ^2^, ^3^ However, these modalities mainly reflect the skeletal muscle status as a static indicator at a somewhat advanced state. Muscle is a highly active metabolic organ, and changes in energy metabolism have been reported to contribute directly to age‐related changes in skeletal muscle in the early stages of sarcopenia.^5^ Because metabolites represent the downstream changes in genome, transcriptome, and proteome expressions, metabolomics is an emerging approach that can reveal inherent omics variation closest to the disease risk/phenotype following recent advances in liquid chromatography–tandem mass spectrometry (LC–MS/MS).^6^ Thus, metabolites may be used as early diagnostic biomarkers of sarcopenia.

Recent studies on metabolomic biomarkers for sarcopenia have reported that some amino acids, lipids, and metabolites related to gut bacterial metabolism are linked with sarcopenia^7^, ^8^, ^9^, ^10^, ^11^, ^12^, ^13^, ^14^; however, metabolomic analysis of sarcopenia is in its early stages.^6^ The use of untargeted metabolomics allows for the observation of a wide range of metabolic features, and recent advances in untargeted metabolomics allow better compound identification due to the use of larger libraries of MS/MS spectra; comparatively, targeted metabolomics provides reliable quantitation.^15^ Thus, untargeted metabolomics offers better opportunities to discover novel metabolites and generate hypotheses, while targeted metabolomics is useful in hypothesis‐driven validation.^6^, ^15^

To identify novel biomarkers of sarcopenia, we conducted metabolomic studies designed in three phases: (1) discovering sphingolipids as sarcopenia‐related metabolomic features in the plasma of an aging mouse model using untargeted metabolomics, (2) exploring sphingolipid expression levels, using targeted metabolomics, for both the aging mouse model of sarcopenia to demonstrate a change of metabolite profiles and muscle cells to demonstrate a dynamic modulation of metabolites during myogenesis, and (3) analysing sphingolipid expression levels using the plasma of elderly men and women with sarcopenia and age‐matched controls in two independent cohorts, to demonstrate the difference in the sphingolipid levels, and their association with sarcopenic parameters. Overall, this study aimed to provide reliable metabolomic biomarkers for sarcopenia.

Sphingolipids, including ceramides (CERs), are bioactive lipids with diverse cellular functions.^16^ CERs, central lipids for sphingolipids synthesis, are mainly synthesized via three canonical pathways: (1) *de novo* synthesis in the endoplasmic reticulum, (2) sphingomyelin (SM) hydrolysis in the cell membrane, and (3) salvaging in the lysosome.^17^ Serine deficiency activates serine palmitoyl‐transferase, which incorporates other amino acids, including alanine, to form deoxysphingolipids via the non‐canonical pathyway.^18^ Mammals have six CER synthases (CERS1–6), each exhibiting characteristic substrate specificity toward acyl‐CoAs with different acyl chain lengths.^19^ Recent studies showed the association of age‐related sphingolipid accumulation in skeletal muscle with muscle mass and function^20^, ^21^ and plasma sphingolipid levels with lean mass (LM)^22^ and gait parameters.^13^ Here, we report across three study phases that some sphingolipid metabolites are potential circulating biomarkers for sarcopenia in men.

## Methods

### Experimental studies using an aging mouse model of sarcopenia and muscle cells

This study used the naturally aging mouse model of sarcopenia.^23^ The Institutional Animal Care and Use Committee of the Asan Institute for Life Sciences (No. 2016‐12‐035) reviewed and approved all animal care and procedures.

Myotube surface area and nuclear fusion index were analysed using MyoCount software (v1.3.1).^12^

### Human cohort study

#### Study participants

Two independent case–control studies were approved by the Institutional Review Board of Veterans Health Service Medical Center (IRB No. 2020‐02‐015) and Asan Medical Center (AMC, IRB No. 2017‐0553) and conducted in compliance with the Helsinki Declaration. Written informed consent was obtained from all participants before enrolment.

For the discovery cohort, participants aged ≥65 years who visited the Division of Endocrinology, Department of Internal Medicine, Veterans Health Service Medical Center (Seoul, Korea) to undergo comprehensive geriatric assessment between August 2020 and March 2021 were enrolled in the ‘Veterans Sarcopenia Study’.^24^ Before the study, all participants completed questionnaires, including medical history, EuroQol Visual Analogue Scale (EQ‐VAS), SARC‐F (Strength, Assistance in walking, Rising from a chair, Climbing stairs, Falls), muscle mass measurement, muscle strength test, and blood sampling.

The validation cohort included patients who visited the AMC (Seoul, Korea) between May 2017 and March 2020 to undergo comprehensive assessment for musculoskeletal disorders. Before the study, all participants completed questionnaires (including medical history), muscle mass measurements, and blood sampling.

#### Assessment of sarcopenia

For the discovery and validation cohorts, body composition was evaluated using bioelectrical impedance analysis (InBody 570, Biospace Co., Seoul, Korea). Appendicular skeletal muscle mass (ASM) was calculated as the sum of the muscle mass in both arms and legs, and the skeletal muscle mass index (SMI) was calculated by dividing ASM by height squared to ensure an objective comparison of muscle mass between participants. The discovery cohort measured muscle strength as handgrip strength (HGS) using a digital hand dynamometer (T.K.K 5401, Takei, Tokyo, Japan). With the participants standing and forearms fully extended in a sideways position away from the body at thigh level, they were instructed to exert maximum grip strength twice with each hand, and the dominant hand was recorded. For the men in this study, low muscle mass was defined as SMI < 7.0 kg/m^2^, and low muscle strength was defined as HGS < 28 kg, according to the consensus of the Asian Working Group for Sarcopenia (AWGS) 2019.^1^ In instances of conflicting results between SMI and HGS, the diagnosis of sarcopenia was determined based on SMI.

Participants with a life expectancy of less than 1 year due to malignancy and those with chronic diseases (heart failure, stroke, Alzheimer's disease, nutrition intake problem, and chronic kidney disease) were excluded. In the discovery cohort, blood samples were collected from 313 eligible participants in the Veterans Sarcopenia Study cohort after excluding ineligible participants.^24^ For each case, controls were matched in a 1:1 ratio for age within a ± 2‐year range. In the validation cohort, controls were matched according to a 2‐year age difference for each case.

#### Metabolome profiling in sample

Metabolites were extracted from samples using liquid–liquid extraction procedures^25^ and were determined using an LC–MS/MS system. Detailed methods are described in the supporting information.

### Statistical analysis

We used the Student's *t*‐test or Mann–Whitney *U* test for continuous variables and the chi‐square test or Fisher's exact test for categorical variables, as needed for comparisons. A *P* value < 0.05 from the Kolmogorov–Smirnov test for normality assessment indicated a non‐normal distribution. The correlation between metabolite levels and relative muscle mass was investigated using Spearman's correlation analysis. Sphingolipid metabolite levels were log‐transformed due to their skewness. Unadjusted logistic regression analyses were performed to generate odds ratios (ORs) with a 95% confidence interval (CI). The area under the receiver‐operating characteristic (ROC) curve (AUROC) was calculated to evaluate the ability of clinical variables and sphingolipid levels to predict sarcopenia. Associations between metabolite levels and sarcopenia parameters (SMI and HGS) were investigated using unadjusted and adjusted multiple linear regression analyses. All statistical analyses were performed using the R 4.2.2 program (R Foundation, Vienna, Austria), with *P* < 0.05 indicating statistical significance.

## Results

### Global metabolome profiling in the plasma of mice

Despite higher body weight, relative muscle mass was significantly lower in the muscles of aged mice than in young mice (*P =* 0.002), indicating age‐related skeletal muscle loss (Table S1).

**Figure 1:**
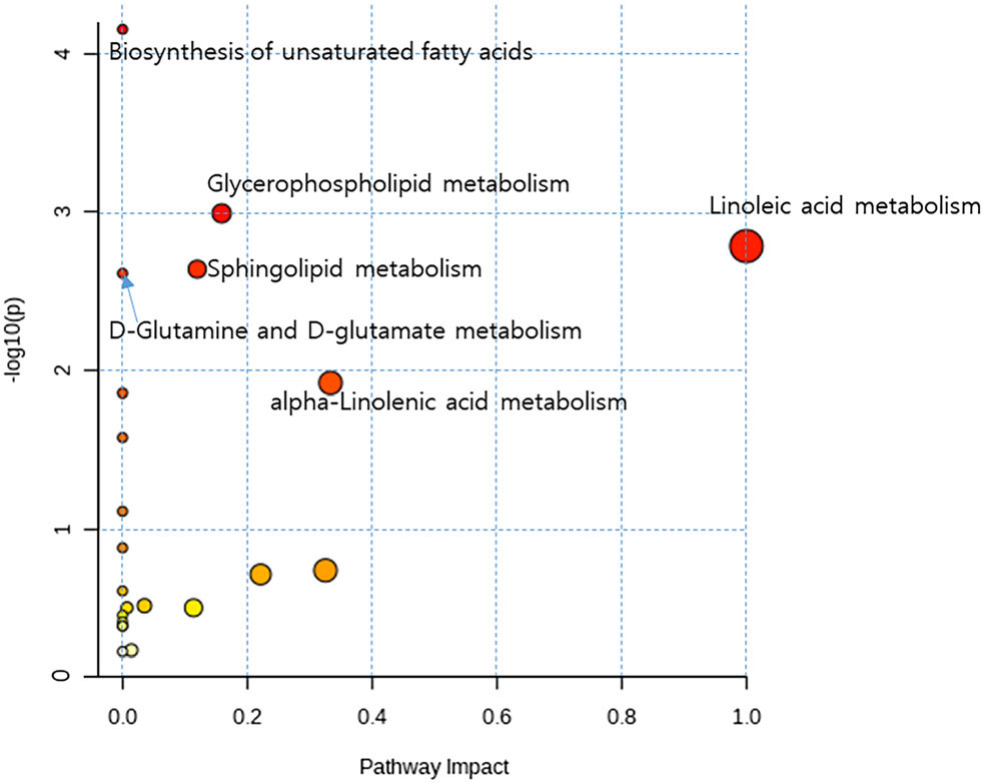
Metabolic pathway analysis for significantly different metabolites between mice from the aging mouse model of sarcopenia and young mice identified by untargeted metabolomics using MetaboAnalyst 5.0 (https://www.metaboanalyst.ca/).

A total of 699 metabolic features were observed in global metabolome profiling; their confidence levels for compound annotation were either level 2 or 3.^26^ A total of 146 metabolites showed statistically significant differences between aged and young mice based on the –log_10_ (*P*‐value) with *P* < 0.05 and FC > 1.2 (Table S2 *)*. Pathway analysis of the 146 significantly changed metabolites showed sphingolipid metabolic pathways potentially related to sarcopenia (Figure 1). Within these pathways, sphingolipid metabolism was selected for further analysis as recent studies showed that the sphingolipid in the skeletal muscle and plasma was associated with age‐related decline in the muscle mass and function^20^, ^21^ and some parameters of sarcopenia, respectively^13^, ^22^.

### Sphingolipid levels in the muscle and plasma of mice

Absolute muscle weight and relative muscle mass were significantly lower in the muscles of aged mice than in young mice (both *P* < 0.05; Table S1). Aged mice tended to have higher plasma CER (24:1) than young mice (*P =* 0.059; Table 1). Plasma CER (24:1) was inversely correlated with relative muscle mass in Spearman's correlation analysis (*r* = −0.522, *P =* 0.034; data not shown). Muscle CER (18:1) content was significantly higher in aged mice than in young mice (*P =* 0.002) and inversely correlated with relative muscle mass (*r* = −0.553, *P =* 0.021; data not shown).

**Table 1:** Sphingolipid levels in mice from the aging mouse model of sarcopenia and young mice

	Plasma concentration (fmol/μL)				Muscle content (fmol/mg)			
	Young (*n* = 10)	Old (*n* = 7)	*P* ^a^	*P* ^b^	Young (*n* = 10)	Old (*n* = 7)	*P* ^a^	*P* ^b^
CER (14:0)	ND	ND			0.4 [0.4; 0.5]	0.5 [0.4; 0.6]	0.142^c^	0.484
CER (16:0)	150.7 ± 36.5	175.1 ± 29.9	0.166	0.385	87.5 [67.0; 104.0]	100.0 [88.0; 113.5]	0.379^c^	0.650
SM (16:0)	26 050.0 [25 400.0; 30 300.0]	29 000.0 [26 800.0; 33 750.0]	0.625^c^	0.983	4171.5 [3185.0; 5233.0]	4914.0 [4042.5; 5027.5]	>0.999^c^	>0.999
CER (18:0)	39.7 ± 11.0	38.5 ± 11.3	0.819	0.819	6350.5 ± 938.9	6883.1 ± 1290.4	0.338	0.580
SM (18:0)	1545.0 [1430.0; 1600.0]	1230.0 [1155.0; 1375.0]	**0.028** ^c^	0.152	4983.7 ± 971.5	5186.6 ± 963.3	0.677	0.738
CER (18:1)	2.6 ± 0.8	2.8 ± 1.1	0.644	0.757	144.4 ± 10.3	181.7 ± 21.0	**<0.001**	**0.002**
SM (18:1)	1098.2 ± 270.8	970.7 ± 263.1	0.349	0.640	490.9 ± 90.7	515.1 ± 114.2	0.632	0.738
CER (20:0)	72.9 ± 17.8	56.6 ± 15.4	0.069	0.251	49.2 ± 14.4	48.4 ± 14.4	0.915	0.915
CER (24:0)	1505.0 [1380.0; 2130.0]	1700.0 [1415.0; 1970.0]	0.962^c^	>0.999	326.9 ± 180.2	225.4 ± 43.7	0.168	0.404
SM (24:0)	110 00.0 [9890.0; 13 600.0]	11 300.0 [10 250.0; 12 850.0]	0.845^c^	>0.999	2360.0 [1671.0; 3533.0]	2107.0 [1608.0; 2190.0]	0.161^c^	0.484
CER (24:1)	1458.9 ± 335.8	2054.3 ± 420.0	**0.005**	0.059	551.9 ± 173.8	690.7 ± 88.8	0.073	0.383
SM (24:1)	29 350.0 [27 800.0; 33 700.0]	34 500.0 [31 950.0; 36 450.0]	0.064^c^	0.233	3528.0 [3196.0; 4883.0]	4345.0 [3353.5; 4465.0]	0.962^c^	>0.999

Data are presented as mean ± SD for sphingolipid levels with normal distribution and as median [IQR] for sphingolipid levels with non‐normal distribution, with significance levels of *P* < 0.05 based on the Kolmogorov–Smirnov test for normality assessment. Bold numbers indicate statistical significance. ND: CER (14:0) plasma levels were below the detection limits of the analytical method.

CER, ceramide; IQR, interquartile range; SD, standard deviation; SM, sphingomyelin.

^a^ The *P*‐value was calculated using the Student's *t*‐test for sphingolipid levels with a normal distribution and the Mann–Whitney *U* test for sphingolipid levels with a non‐normal distribution.

^b^ *P*‐value after adjusting for multiple testing corrections using the false discovery rate (FDR) method.

^c^ *P*‐value < 0.05 based on the Kolmogorov–Smirnov test for normality assessment, indicating non‐normal distribution of data.

### Sphingolipid levels in differentiating muscle cells

**Figure 2:**
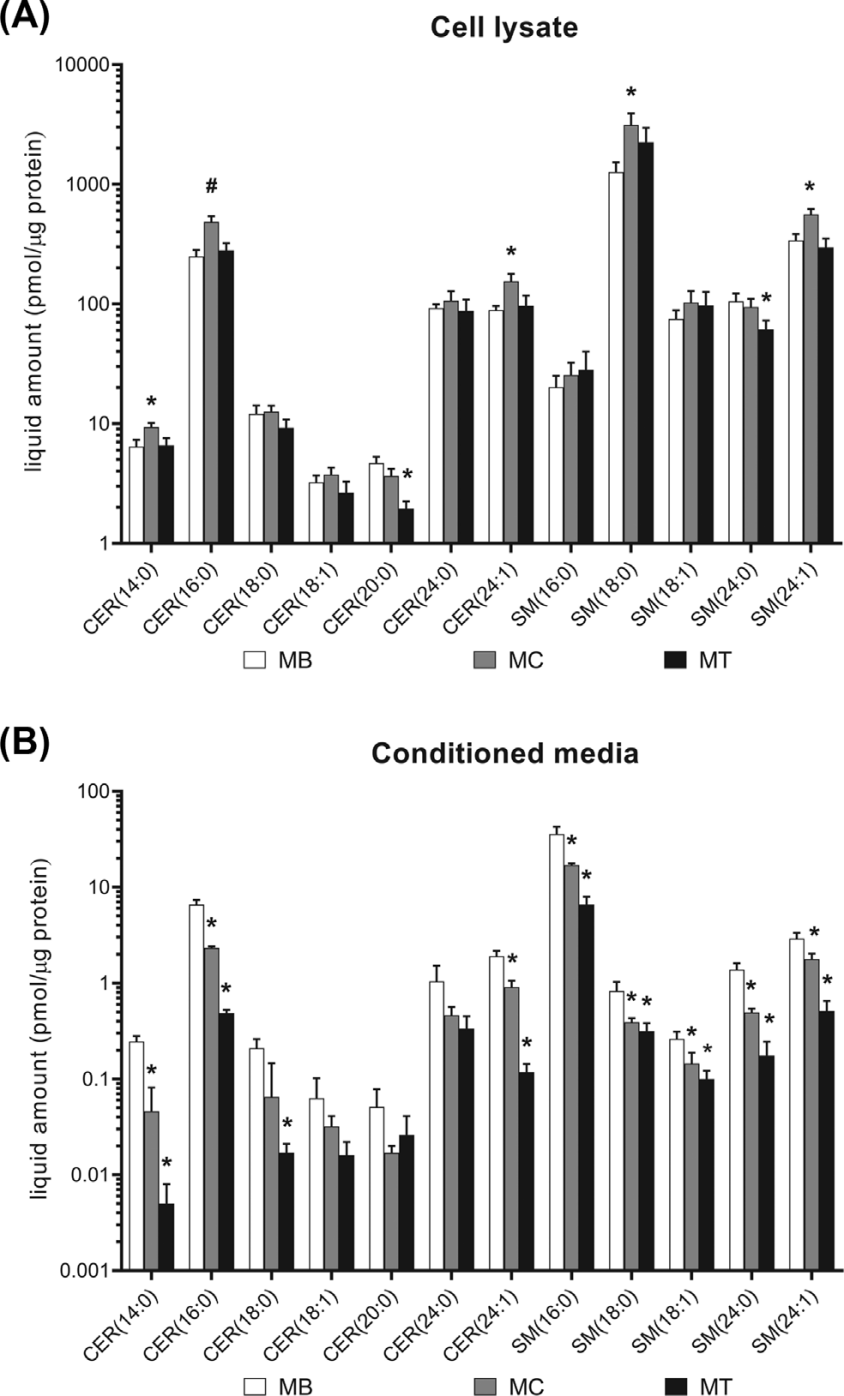
Changes in sphingolipid levels during myoblast differentiation. Sphingolipid levels in cell lysate (A) and conditioned media (B) of myoblasts (MBs), myocytes (MCs), and myotubes (MTs). Significant changes in the level of each sphingolipid species in MCs and MTs compared with those in MBs are shown as *P < 0.05 and ^#^ *P* < 0.001. The error bar represents the standard deviation. *P* values were calculated using the Mann–Whitney *U* analysis.

Changes in sphingolipid levels in the cell lysate and CM of differentiating muscle cells were also explored. CER (14:0), CER (16:0), CER (24:1), SM (18:0), and SM (24:1) levels in cell lysates were significantly higher in MC than MB but not significantly different between MT and MB. CER (20:0) and SM (24:0) levels were significantly lower in MT than MB (all *P* < 0.05; Figure 2A). Alternatively, most of the CERs and SMs in CM were significantly lower at the later stages of differentiation (MC vs. MB and MT vs. MB, all *P* < 0.05; Figure 2B).

### Characteristics of study participants in the discovery cohort

The main characteristics of the 144 men in the discovery cohort (72 men with sarcopenia and 72 age‐matched controls) from the Veterans Sarcopenia Study are presented in Table 2. There were no significant differences in age between the case and control groups (*P* = 0.989). The weight, height, and BMI of the sarcopenia group were significantly lower than in the control group (all *P* < 0.001). Muscle mass parameters (lean mass, ASM, and SMI) and HGS in the case group were significantly lower than in the control group (all *P* < 0.001). Weight was highly correlated with SMI following Spearman's correlation analysis (*r* = 0.871, *P* < 2.2E‐16; data not shown). There were no significant differences in the chair stand test score, drinking and exercise habits, or prevalence of hypertension and diabetes between the case and control groups (all *P* > 0.05).

**Table 2:** Baseline characteristics of men in the discovery cohort (*n* = 144)

Disease	Control (*n* = 72)	Sarcopenia (*n* = 72)	*P*
Age (years)	74.0 [72.0; 76.0]	74.0 [72.0; 76.0]	0.989
Weight (kg)	72.7 [66.5; 79.5]	56.2 [51.2; 59.1]	<0.001
Height (cm)	167.8 ± 4.8	163.0 ± 5.1	<0.001
BMI (kg/m^2^)	26.1 [24.1; 27.9]	21.1 [19.4; 22.6]	<0.001
Smoking, *N* (%)			0.002
Ex‐smoker	57 (79.2%)	42 (58.3%)	
Non‐smoker	12 (16.7%)	13 (18.1%)	
Current smoker	3 (4.2%)	17 (23.6%)	
Drinking, *N* (%)			0.174
No alcohol	34 (47.2%)	41 (56.9%)	
Alcohol <1/week	20 (27.8%)	19 (26.4%)	
Alcohol 1–2/week	12 (16.7%)	4 (5.6%)	
Alcohol ≥3/week	6 (8.3%)	8 (11.1%)	
Exercise, *N* (%)			0.397
No exercise	6 (8.3%)	12 (16.7%)	
Exercise <1/week	4 (5.6%)	6 (8.3%)	
Exercise 1–2/week	12 (16.7%)	10 (13.9%)	
Exercise ≥3/week	50 (69.4%)	44 (61.1%)	
Hypertension, *N* (%)	50 (69.4%)	37 (52.1%)	0.051
Diabetes, *N* (%)	60 (95.8%)	64 (90.1%)	0.314
FM (kg)	21.6 [16.9; 23.9]	14.1 [11.2; 17.8]	<0.001
pFM (%)	28.1 ± 6.2	25.0 ± 6.6	0.003
EQ‐VAS	75.0 [60.0; 85.0]	65.0 [50.0; 77.5]	<0.001
SARC‐F	0.0 [0.0; 1.0]	1.0 [0.0; 2.0]	0.005
HGS (kg)	34.6 ± 7.0	28.5 ± 5.6	<0.001
Chair stand up test (s)	7.0 [6.0;9.0]	7.0 [5.0;8.0]	0.874
LM (kg)	49.6 [47.7; 52.4]	39.8 [38.0; 41.6]	<0.001
ASM (kg)	22.4 [21.4; 23.9]	16.9 [16.1; 18.2]	<0.001
SMI (kg/m^2^)	8.0 [7.6; 8.4]	6.5 [6.2; 6.6]	<0.001

Data are presented as mean ± SD for normally distributed continuous variables, median [IQR] for non‐normally distributed continuous variables with significance levels of *P* < 0.05 based on the Kolmogorov–Smirnov test for normality assessment, or number (%) for categorical variables. We used the Student's *t*‐test for continuous variables when normality was satisfied and the Mann–Whitney *U* test for a non‐normal distribution. For categorical variables, we used the chi‐square or Fisher's exact test. Bold numbers indicate statistically significant values.

ASM, appendicular skeletal muscle mass; BMI, body mass index; EQ‐VAS, EuroQol Visual Analogue Scale; FM, fat mass; HGS, hand grip strength; IQR, interquartile range; LM, lean mass; pFM, per cent fat mass; SARC‐F, Strength, Ambulation, Rising from a chair, stair Climbing, and history of Falling; SD, standard deviation; SMI, skeletal muscle mass index.

The main characteristics of the 72 women in the discovery cohort (36 women with sarcopenia and 36 age‐matched controls) from the Veterans Sarcopenia Study are presented in Table S3. Muscle mass parameters (lean mass, ASM, and SMI), but not HGS, were significantly lower in the case group than the control group (all *P* < 0.001).

### Association between sphingolipid metabolites in human plasma and sarcopenia in the discovery cohort

Plasma SM (16:0), CER (24:1), and SM (24:1) levels were significantly higher in men with sarcopenia than in the control group (all *P* < 0.05; Table 3). Contrastingly, there were no significant differences in the sphingolipid levels in women with or without sarcopenia in the discovery cohort (Table S4). Higher long‐chain and very‐long‐chain dihydroceramide levels were observed in men with sarcopenia via the canonical pathway, although the difference between the two groups was not statistically significant (Table S5). Additionally, there was no significant difference between the two groups of amino acid levels (serine, alanine, and glycine) and deoxyCER levels via the non‐canonical pathway.

**Table 3:** Plasma sphingolipid levels of men in the discovery cohort based on targeted metabolome profiling (*n* = 144).

	Control (*n* = 72)	Case (*n* = 72)	log2(FC)	*P* ^a^	*P* ^b^
CER (14:0) (μM)	13.6 [11.3;15.2]	13.6 [11.4;16.6]	−0.066	0.740^c^	0.807
CER (16:0) (μM)	64.1 ± 15.9	69.6 ± 18.2	−0.121	0.052	0.154
SM (16:0) (μM)	40 641.7 ± 6461.3	45 047.3 ± 8671.9	−0.148	0.001	0.003
CER (18:0) (μM)	35.0 [29.1;41.6]	38.3 [27.4;47.1]	−0.039	0.592 ^c^	0.763
SM (18:0) (μM)	23 458.6 [20 875.5; 26 159.6]	24 353.6 [20 977.2; 29 426.1]	−0.098	0.097 ^c^	0.233
CER (18:1) (μM)	7.0 [5.7; 8.5]	7.0 [5.2; 8.8]	−0.007	0.924 ^c^	0.924
SM (18:1) (μM)	5341.0 [4408.0;6126.4]	5312.2 [4152.9;6590.9]	−0.059	0.584 ^c^	0.763
CER (20:0) (μM)	92.8 [77.9;108.5]	92.5 [77.8;117.3]	−0.076	0.636 ^c^	0.763
CER (24:0) (μM)	1682.0 [1445.8;1995.8]	1891.3 [1438.7;2318.8]	−0.149	0.122 ^c^	0.243
SM (24:0) (μM)	13 430.5 [10 920.2; 15 545.4]	14 214.1 [11 524.3; 16 040.8]	−0.046	0.401 ^c^	0.688
CER (24:1) (μM)	573.0 [463.0;653.8]	652.8 [544.0;835.9]	−0.277	0.001 ^c^	0.003
SM (24:1) (μM)	31 586.6 [27 603.5; 35 510.3]	35 924.8 [31 117.2; 40 928.9]	−0.183	<0.001 ^c^	0.003

Data are presented as mean ± SD for sphingolipid levels with normal distribution and as median [IQR] for sphingolipid levels with non‐normal distribution, with significance levels of *P* < 0.05 based on the Kolmogorov–Smirnov test for normality assessment. Bold numbers indicate statistically significant values.

CER, ceramide; FC, fold change; IQR, interquartile range; SD, standard deviation; SM, sphingomyelin.

^a^ *P*‐value was calculated using Student's *t*‐test for sphingolipid levels with a normal distribution and the Mann–Whitney *U* test for sphingolipid levels with a non‐normal distribution.

^b^ *P*‐value after adjusting for multiple testing corrections using the false discovery rate (FDR) method.

Univariate analysis showed that increasing SM (16:0) levels (per SD) tended to increase the odds of low muscle strength (OR = 1.43, 95% CI: 0.99–2.08; *P* = 0.058). The AUROC of SM (16:0) for low muscle strength in men was 0.600 (95% CI: 0.501–0.699). Increasing SM (16:0), CER (24:1), and SM (24:1) levels (per SD) significantly increased the odds of low muscle mass with ORs of 1.76 (95% CI: 1.23–2.52), 2.04 (95% CI: 1.39–3.00), and 1.90 (95% CI: 1.31–2.77), respectively (all *P* < 0.01; Table 4). The AUROC of SM (16:0), CER (24:1), and SM (24:1) for low muscle mass in men was 0.647 (95% CI: 0.557–0.737), 0.669 (95% CI: 0.581–0.757), and 0.670 (95% CI: 0.582–0.759), respectively.

**Table 4:** Association of sphingolipid metabolites with low muscle strength and low muscle mass in the discovery cohort male (*n* = 144)

	OR (95% CI)	*P*	AUC (95% CI)	*P*
Low muscle strength				
SM (16:0) per 1SD	1.43 (0.99–2.08)	0.058	0.600 (0.501–0.699)	0.049
CER (24:1) per 1SD	1.24 (0.86–1.77)	0.251	0.571 (0.464–0.677)	0.193
SM (24:1) per 1SD	1.23 (0.85–1.76)	0.270	0.563 (0.463–0.663)	0.214
Low muscle mass				
SM (16:0) per 1SD	1.76 (1.23–2.52)	0.002	0.647 (0.557–0.737)	0.001
CER (24:1) per 1SD	2.04 (1.39–3.00)	<0.001	0.669 (0.581–0.757)	<0.001
SM (24:1) per 1SD	1.90 (1.31–2.77)	0.001	0.670 (0.582–0.759)	<0.001

Sphingolipid metabolite levels were log‐transformed because of their skewed distribution. HGS cutoff points for low muscle strength were <28 kg. SMI cutoff points for low muscle mass were <7.0 kg/m^2^. Bold numbers indicate statistically significant values.

95% CI, 95% confidence interval; AUC, area under the receiver‐operating characteristic (ROC) curve; CER, ceramide; HGS, hand grip strength; OR, odds ratio; SD, standard deviation, SM, sphingomyelin.

SM (16:0), CER (24:1), and SM (24:1) were significantly inversely associated with SMI (all *P* < 0.05). After adjusting for weight, the statistical significance of the inverse association of CER (24:1) with SMI remained, as shown in Table 5. SM (16:0) tended to be inversely associated with HGS (*P* = 0.096). Multivariate analysis showed a significant association between CER (24:1) and low muscle mass.

**Table 5:** Association between sphingolipid metabolite levels and HGS and SMI in the discovery cohort males (*n* = 144)

	Univariate				Multivariate				Adjustment for weight			
	β^a^	SE	β^b^	*P*	β^a^	SE	β^b^	*P*	β^a^	SE	β^b^	*P*
SMI												
SM (16:0) per 1SD	−0.205	0.075	−0.223	0.007	−0.129	0.079	−0.141	0.103	0.016	0.039	0.017	0.681
CER (24:1) per 1SD	−0.258	0.074	−0.281	0.001	−0.212	0.079	−0.231	0.008	−0.101	0.037	−0.110	0.007
SM (24:1) per 1SD	−0.187	0.075	−0.204	0.014					0.006	0.038	0.006	0.879
HGS												
SM (16:0) per 1SD	−0.977	0.583	−0.139	0.096	−0.977	0.583	−0.139	0.096	−0.095	0.544	−0.014	0.862
CER (24:1) per 1SD	−0.444	0.587	−0.063	0.451					0.216	0.533	0.031	0.686
SM (24:1) per 1SD	−0.536	0.587	−0.076	0.363					0.260	0.538	0.037	0.629

Sphingolipid metabolite levels were log‐transformed because of their skewed distribution. Multivariate analysis was selected using a backward elimination method. Bold numbers indicate statistically significant values.

CER, ceramide; HGS, hand grip strength; SM, sphingomyelin; SMI: skeletal muscle mass index.

^a^ Unstandardized coefficient.

^b^ Standardized coefficient.

### Effect of CER treatment on myogenesis in vitro

Treatment with CER (16:0), CER (18:0), and CER (24:1) significantly decreased the MT area and fusion index (Figure S1). Treatment with CER (16:0), CER (18:0), and CER (24:1) also decreased the viability of muscle cells.

### Association between sphingolipid metabolites in human plasma and sarcopenia in the validation cohort

The main characteristics of the 164 participants in the validation cohort (36 cases and 128 age‐matched controls) from the AMC are presented in Table S6. Muscle mass parameters (lean mass, ASM, and SMI) in the case group were significantly lower than in the control group (all *P* < 0.001). HGS measurements in 74 of 164 (45.1%) participants in the validation cohort [control group: 50 of 128 (39.1%) and control group: 24 of 36 (66.7%)] revealed that HGS in the case group was significantly lower than in the control group (*P* = 0.013). The sarcopenia group also had significantly higher SM (16:0), CER (24:1), and SM (24:1) levels than the control group (all *P* < 0.005). Increasing CER (24:1) and SM (24:1) levels (per SD) significantly increased the odds of low muscle mass with ORs of 1.47 (95% CI: 1.01–2.15) and 2.85 (95% CI: 1.82–4.47), respectively (data not shown).

### The predictive ability of the Sphingolipid (SphL) score for sarcopenia in both the discovery and validation cohorts

**Figure 3:**
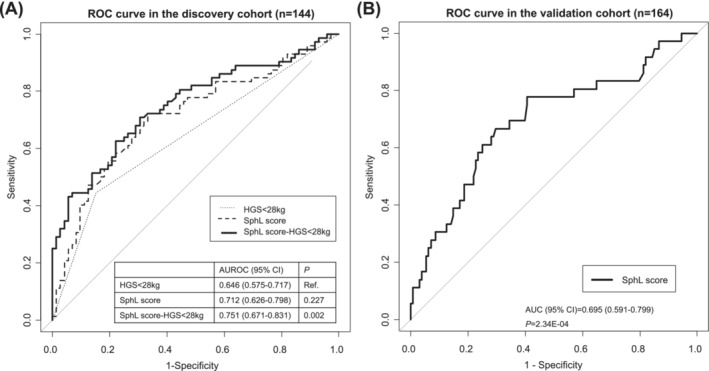
The ROC of the SphL score for detecting sarcopenia of men in the discovery cohort (*n* = 144) and those in the validation cohort (*n* = 164). AUROC, area under the receiver‐operating characteristic (ROC) curve; HGS, hand grip strength; SphL score = 0.004 + 0.594*log (CER (24:1) SD) + 0.397*log (SM (16:0) SD); 95% CI, 95% confidence interval.

The SphL score, a diagnostic regression equation for sarcopenia based on multivariate linear regression analysis of CER (24:1) and SM (16:0) with SMI in the discovery cohort, was calculated as follows: SphL score = 7.216–0.212*log (CER (24:1) SD)‐0.129*log (SM (16:0) SD). The corresponding ROC curve of the SphL score using a combination of CER (24:1) and SM (16:0) levels had an AUC of 0.712 (95% CI: 0.626–0.798) in the discovery cohort (Figure 3). The addition of the SphL score to low muscle strength (HGS < 28 kg) as a predictor of sarcopenia improved the AUC by 16.3%, from 0.646 (95% CI: 0.575–0.717; HGS only) to 0.751 (95% CI: 0.671–0.831, *P* = 0.002; HGS + SphL). The AUC of the SphL score was 0.695 (95% CI: 0.591–0.799) in the validation cohort. When analysing the 74 participants with HGS data in the validation cohort, the SphL score was significantly predictive of sarcopenia (AUC: 0.678; 95% CI: 0.541–0.816), although the HGS was not (AUC: 0.553; 95% CI: 0.470–0.636).

## Discussion

In this study, we aimed to provide reliable metabolomic biomarkers for sarcopenia using mouse and cell models and two cohorts of human participants. Non‐targeted metabolome profiling in the plasma of mice from an aging mouse model of sarcopenia showed that sphingolipid metabolism was related to sarcopenia (Figure 1). This result was confirmed by higher plasma CER (C24:1) levels and muscle CER (C18:1) content in aged mice, and both of plasma CER (24:1) levels and muscle CER (18:1) contents were inversely associated with relative muscle mass (Table 1). Moreover, most of CERs and SMs in CM decreased during muscle cell differentiation (Figure 2). Quantitation of sphingolipids from human plasma showed that the SM (16:0), CER (24:1), and SM (24:1) levels were higher in men with sarcopenia than in control participants (Table 3). However, there were no significant differences in sphingolipid metabolites in women with or without sarcopenia. ROC analysis showed the association of the plasma SM (16:0) level in men with lower HGS, which reflects muscle strength (Table 4). ROC analysis also showed the association of plasma SM (16:0), CER (24:1) and SM (24:1) in men with lower SMI, which reflects muscle mass (Table 4). The SphL score, a model for predicting sarcopenia using CER (24:1) and SM (16:0) levels, was significantly predictive for sarcopenia in men in two independent cohorts (Figure 3). The addition of SphL score to HGS in the discovery cohort significantly improved the predictive performance of the latter for sarcopenia, as shown by a 16.3% increase in AUC (Figure 3). To the best of our knowledge, our study is the first to suggest that SM (16:0), reflecting low muscle strength and low muscle mass, and CER (24:1), reflecting low muscle mass, could serve as biomarkers for sarcopenia in men.

The processes underlying sarcopenia pathogenesis are not fully understood and may involve multiple factors, such as a decline in mitochondrial and cellular protein homeostasis.^27^ Importantly, evidence shows that sphingolipid metabolism perturbations are associated with sarcopenia development and progression of sarcopenia.^13^, ^17^, ^20^, ^22^, ^28^, ^29^, ^30^, ^31^, ^32^, ^33^ For example, in vitro experimental studies suggest that CERs negatively regulate myogenic differentiation through their involvement in apoptosis, cellular stress, and cell growth arrest.^17^, ^29^, ^30^, ^31^, ^33^ Additionally, some in vivo experimental studies also demonstrate that sphingolipid metabolism could be involved in the development of sarcopenia and muscle atrophy.^17^, ^22^ Furthermore, sphingolipids contribute to muscle aging, and reducing them prevents age‐related decline in muscle mass while enhancing strength and exercise capacity^20^, ^21^ by affecting mitochondria and protein homeostasis.^18^, ^21^ In humans, the muscle CER (16:0) content in a small sample of men (*n* = 19) was inversely correlated with lean leg mass,^22^ while plasma CER (16:0) levels in 340 clinically normal participants aged 70 years and older were inversely associated with gait parameters, including gait speed.^13^ Overall, these findings suggest that sphingolipids may play a direct role in sarcopenia and that sphingolipid metabolites may be useful as biomarkers of sarcopenia.

In the present study, plasma levels of three sphingolipid metabolites, SM (16:0), CER (24:1), and SM (24:1), were higher in men with sarcopenia than those without. Although not statistically significant, the higher long‐ and very‐long‐chain dihydroCER and serine levels might suggest that the canonical rather than the non‐canonical pathway is associated with sarcopenia. The association between SM (16:0) and HGS was similar to that in a previous study, which showed an inverse association between CER (16:0) and gait speed.^13^ Consistent with our findings of an inverse association between SM (16:0), CER (24:1), and SM (24:1) and SMI, another recent study found that increased plasma CER (16:0), CER (24:0), SM (16:0), and SM (24:1) levels were a defining feature of the mouse and human cancer cachexia models; authors suggested that these sphingolipids may contribute to tissue wasting and skeletal muscle atrophy by inhibiting the anabolic signals.^34^ The disparities between the two groups in humans, especially weight, would be suggested as a discriminator between sarcopenia and age‐matched controls. However, (1) statistically persistent significance in the negative association of CER (24:1) level with SMI after adjustment for weight, (2) tendency for higher CER (24:1) levels in the aging mouse model of sarcopenia regardless of higher weight, and (3) no significant difference in the prevalence of comorbidities were observed; thus, differences in sphingolipid levels could be considered as biomarkers of disease status. Our prediction model for sarcopenia, calculated by combining CER (24:1) and SM (16:0) levels, significantly predicted sarcopenia in both cohorts. Adding a model for sarcopenia by combining CER (24:1) and SM (16:0) to HGS improved the discriminatory performance of HGS in the discovery cohort. If confirmed by another large independent cohort, these findings might support using CER (24:1) and SM (16:0) as biomarkers for sarcopenia.

Consistent with our findings, recent studies have shown that reducing sphingolipids, such as CER (24:1) and CER (18:1), accumulating in mouse skeletal muscle with age prevented age‐related decline in muscle mass while enhancing strength and exercise capacity.^20^, ^21^ In line with findings of CERs as negative regulators of myogenic differentiation,^17^, ^29^, ^30^, ^31^, ^33^ some CERs and all SMs in CM decreased during muscle cell differentiation. Previous studies showed that inhibiting sphingolipid synthesis can mitigate age‐related muscle decline and improve muscle function.^20^, ^21^ However, while our study focuses on identifying diagnostic biomarkers, the detrimental cellular effects on myogenesis and cell viability by CER (16:0), CER (18:0), and CER (24:1) observed in the present study and previous studies suggest the role of sphingolipids as a cause of sarcopenia. We recognize the necessity for further research to identify the specific CERs that are not only indicative of sarcopenia but may also serve as targets for treatment. This dual role of CERs as both biomarkers and therapeutic targets warrants deeper exploration in the context of age‐related sarcopenia.

Consistent with previous studies showing metabolic differences between sexes,^35^ we found statistically significant differences in sphingolipid metabolism according to sarcopenia status in men but not women. The target metabolome was selected based on the result generated by untargeted metabolomics using male mice only, which may result in metabolite changes more relevant to men. Untargeted metabolomics for female mice would be necessary to find female‐relevant metabolome changes. Additionally, a broad range of target metabolomes must be explored to find common biomarkers for both men and women. Another conflicting finding occurred in the mouse studies, where, compared with young mice, aged mice had higher CER (24:1) but lower SM (18:0) plasma levels. Interestingly, some studies show that supplementation of SM in mice actually increases muscle weight^36^ and extends swimming time,^36^, ^37^ which suggests that there may be SM acyl chain length‐specific effects on muscle in mice. Nonetheless, this change was not seen in the human cohort, where the plasma SM (18:0) level tended to be higher in men with sarcopenia than those without, suggesting that sphingolipids may have species–specific effects on muscle in mice and humans.^19^ Increased plasma SM (16:0) and CER (24:1) levels in men with sarcopenia reflected increased CER species with fatty acid acyl chain length preferences toward long (C16:0) and very‐long chains (C24:0 and C24:1), which CERS6 and CERS2, respectively, produce.^21^ These results align with previous studies that applied transcriptomic analysis of muscle biopsies from aged individuals and patients with muscle disorders.^21^ Another study reported the reduced expression of CERS1 and CERS5 in human skeletal muscles with aging.^38^ Moreover, the inhibition of CERS1 in mice skeletal muscles and primary human muscle cells increased CER (16:0) and CER (24:1).^38^

A major strength of our study is that it included a relatively large number of elderly men with matched controls and validation in an independent cohort. Additionally, animal and muscle cell experiments strengthen the robustness of the study's findings and its validity. However, this study has some limitations. First, the causal relationship between identified sphingolipids and sarcopenia cannot be inferred because of the cross‐sectional study design. Second, age‐matched animal models of sarcopenia would be preferable to the differently aged animals used in this study. We aimed to find the circulating biomarkers for sarcopenia in humans, so we could not measure sphingolipid metabolites in various tissues, including oxidative and glycolytic muscles and other organs. Third, the accuracy of BIA, which was used to diagnose sarcopenia as recommended by the AWGS, had limitations related to equipment accuracy and assessment conditions.^39^ Despite this concern, the high diagnostic accuracy of BIA, aligning with dual‐energy X‐ray absorptiometry in a substantial subset of the validation cohort (62 of 164, 37.8%) by 93.5%, with a sensitivity of 100.0% (17 of 17) and specificity of 91.1% (41 of 45), suggested that BIA could be used to diagnose sarcopenia. Fourth, we did not measure HGS in all of the validation cohort participants; thus, the results relating to HGS might not be representative. Accumulating evidence shows the clinical relevance of functional parameters over muscle mass; therefore, recent guidelines emphasize the importance of measuring muscle strength or physical performance over muscle mass.^1^, ^2^ However, the association of SM (16:0) with HGS and risk of low muscle strength in the discovery cohort, and that of CER (16:0) with gait speed in another study,^13^ might support that sphingolipids are related to not only muscle mass but also muscle strength or physical performance. We did not define a specific tissue, including muscle types, responsible for the secretion of the sphingolipid metabolites in the plasma, although recent publications related to the specific increases of sphingolipid species in skeletal muscle or reduced/compensatory expression of CERS with aging might support our result.^21^, ^38^

Our study demonstrates that SM (16:0), reflecting low muscle strength and low muscle mass, and CER (24:1), reflecting low muscle mass, are potential circulating biomarkers for sarcopenia in men. Further research on sphingolipid metabolites is required to confirm these results and provide additional insights into the metabolomic changes relevant to the pathogenesis and diagnosis of sarcopenia.

## Conflict of interest

Je Hyun Seo, Jung‐Min Koh, Han Jin Cho, Hanjun Kim, Young‐Sun Lee, Su Jung Kim, Pil Whan Yoon, Won Kim, Sung Jin Bae, Hong‐Kyu Kim, Hyun Ju Yoo, and Seung Hun Lee declare that they have no conflict of interest.

## Funding

This study was supported by the Asan Institute for Life Sciences Grant (grant number: 2023IP0041) and the National Research Foundation of Korea (NRF) grant, funded by the Korean government (Ministry of Science and ICT; grant numbers: 2022R1C1C1002929, 2022R1A2C1007901, and 2022R1A2C1003661). The authors of this manuscript certify that the study complies with the ethical guidelines for authorship and publishing in the *Journal of Cachexia, Sarcopenia, and Muscle*.^40^

## Supporting information


**Figure S1.** Effects of ceramide (CERs) on myogenesis. (A) C2C12 myoblasts were incubated with 0 or 2.5 μM CERs for 3 days. Myotubes were stained with an anti‐myosin (Skeletal, Fast) heavy chain antibody. Photographs of immunofluorescent images and quantitative results of myosin heavy chain positive area and fusion index are shown. Scale bars: 200 μm. (B) C2C12 myoblasts were incubated with 0 or 2.5 μM CERs for 2 days. Cell viability was determined using a Cell Counting Kit‐8. Each bar represents the mean ± standard deviation (SD) (*n* = 6).

**Table S1.** Baseline characteristics of young and old mice from the aging mouse model of sarcopenia.
**Table S3**. Baseline characteristics of women in the discovery cohort (*n* = 72).
**Table S4**. Plasma sphingolipid levels of women in the discovery cohort based on targeted metabolome profiling (n = 72).
**Table S5**. Plasma dihydroceramide levels and amino acid (alanine, serine, glycine) and deoxyceramide levels in men in the discovery cohort based on targeted metabolome profiling (*n* = 144).
**Table S6**. Baseline characteristics of men in the validation cohort (*n* = 164).

**Table S2.** Metabolites identified by untargeted metabolomics in young mice and mice from the aging mouse model of sarcopenia.

## Data Availability

Data generated/analysed during the study are available from the corresponding author upon reasonable request.
