# Supplementary material for: Sphingolipid metabolites as potential circulating biomarkers for sarcopenia in men
Source: J Cachexia Sarcopenia Muscle. 2024 Sep 4;15(6):2476–86. doi: 10.1002/jcsm.13582 (PMC11634516; doi:10.1002/jcsm.13582)
Supplement: Supplementary file 2 — Table S1. Baseline characteristics of young and old mice from the aging mouse model of sarcopenia. Table S3. Baseline characteristics of women in the discovery cohort (n = 72). Table S4. Plasma sphingolipid levels of women in the discovery cohort based on targeted metabolome profiling (n = 72). Table S5. Plasma dihydroceramide levels and amino acid (alanine, serine, glycine) and deoxyceramide levels in men in the discovery cohort based on targeted metabolome profiling (n = 144). Table S6. Baseline characteristics of men in the validation cohort (n = 164). [file JCSM-15-2476-s003.docx]

**Sphingolipid metabolites as potential circulating biomarkers for sarcopenia in men**

Je Hyun Seo,^1,a^ Jung-Min Koh,^2,a^ Han Jin Cho,^3^ Hanjun Kim,^3^ Su Jung Kim,^4^ Pil Whan Yoon,^5^ Won Kim,^6^ Sung Jin Bae,^7^ Hong-Kyu Kim,^7^ Hyun Ju Yoo,^4,b^ Seung Hun Lee^2,b^

^1^Veterans Medical Research Institute, Veterans Health Service Medical Center, Seoul, South Korea

^2^Division of Endocrinology and Metabolism, Asan Medical Center, University of Ulsan College of Medicine, Seoul, South Korea

^3^Biomedical Research Center, Asan Institute for Life Sciences, Asan Medical Center, Seoul, South Korea

^4^Department of Convergence Medicine, Asan Institute for Life Sciences, Asan Medical Center, University of Ulsan College of Medicine, Seoul, South Korea

^5^Department of Orthopedic Surgery, Seoul Now Hospital, Anyang, South Korea

^6^Department of Rehabilitation Medicine, Asan Medical Center, University of Ulsan College of Medicine, Seoul, South Korea

^7^Health Screening and Promotion Center, Asan Medical Center, University of Ulsan College of Medicine, Seoul, South Korea

^a^JHS and J-MK are joint first authors.

^b^HJY and SHL are joint corresponding authors.

Corresponding authors:

Hyun Ju Yoo, Ph.D.

Department of Convergence Medicine, Asan Institute for Life Sciences, Asan Medical Center, University of Ulsan College of Medicine, Seoul, South Korea

Tel: +82-2-3010-4029; E-mail: [yoohyunju@amc.seoul.kr](mailto:yoohyunju@amc.seoul.kr)

Seung Hun Lee, M.D., Ph.D.

Division of Endocrinology and Metabolism, Department of Medicine, Asan Medical Center, University of Ulsan College of Medicine, 88 Olympic-Ro 43 gil, Songpa-gu, Seoul 05505, Korea

Tel: +82-2-3010-5666; Fax: +82-2-3010-6962; E-mail: [hun0108@amc.seoul.kr](mailto:hun0108@amc.seoul.kr)

**Supporting information**

**Methods**

**Experimental studies**

***Aging mouse model of sarcopenia***

Since aging is the main risk factor for sarcopenia, the present study used the natural aging mouse model of sarcopenia, which has been widely used in the study of sarcopenia^23^. Male C57BL/6 mice were purchased from the Korea Research Institute of Bioscience and Biotechnology (Daejeon, South Korea). Most naturally aging mouse models of sarcopenia use mice over 18 months of age^23^; therefore, mice aged 20 and 19 months, reflecting old age in humans, were used for untargeted and targeted metabolomics, respectively. Mice aged 3 months and 10 months, reflecting young age in humans, were used as controls for untargeted and targeted metabolomics, respectively. All mice were housed in an environmentally controlled, pathogen-free room with a 12 h/12 h light/dark cycle and free access to laboratory chow and water. After 4 h of fasting, mice were euthanized by cardiac puncture under anesthesia induced by an intraperitoneal injection of 50 mg/kg Zoletil 50 (Virbac, Carros, France) and 10 mg/kg Rompun (Bayer Korea, Seoul, South Korea). Relative muscle mass (%) was defined as the weight of the isolated muscle as a percentage of total body weight. All animal care and procedures were reviewed and approved by the Institutional Animal Care and Use Committee of the Asan Institute for Life Sciences (No. 2016-12-035).

***Differentiation of muscle cells***

Murine C2C12 myoblasts (MBs) were purchased from American Type Culture Collection (ATCC, Rockville, MD, USA) and cultured in Dulbecco’s modified Eagle’s medium (DMEM) containing 10% fetal bovine serum (Gibco, Grand Island, NY, USA), 100 U/mL penicillin, and 0.1 mg/mL streptomycin in a humidified CO_2_ incubator at 37 °C. C2C12 MBs were differentiated in DMEM containing 2% horse serum for 2 d (myocytes, MCs) or 6 d (myotubes, MTs). MBs, MCs, or MTs were incubated in serum-free DMEM for 24 h. The 24 h-conditioned medium (CM) was collected, and total cell lysates were prepared in a radioimmunoprecipitation (RIPA) buffer.

***Effect of ceramide (CER) treatment on myogenesis in vitro***

CER (16:0), CER (18:0), and CER (24:1) were purchased from Cayman Chemical Company (Ann Arbor, MI, USA) and dissolved in dimethyl formamide. To examine the effects of CERs on myogenesis, C2C12 myoblasts were seeded at 25,000 cells/well in a 24-well plate. After 24 h, cells were incubated in DMEM, supplemented with 2% horse serum containing 2.5 μM of CERs for 72 h. The cultures were fixed in 4% paraformaldehyde for 15 min, permeabilized in phosphate-buffered saline (PBS) containing 10 mM sodium citrate and 0.1% Tritone X-100 for 10 min, and blocked with 2% bovine serum albumin for 1 h. The cultures were probed with an anti-myosin heavy chain (Skeletal, Fast) antibody (Sigma-Aldrich, St. Louis, MO, USA) at 4 °C overnight and then incubated with Alexa 555-conjugated anti-mouse IgG for 1 hr. Nuclei were counterstained with 4’,6-diamidino-2-phenylindole. Fluorescence images were obtained using an Axio Imager Microscope (Carl Zeiss, Oberkochen, Germany) and were analyzed using MyoCount software (v1.3.1) for myotube area and fusion index^12^.

To examine the effects of CERs on cell viability, C2C12 myoblasts were seeded at 3,000 cells/well in 96-well plates. After 24 h, cells were incubated in DMEM, supplemented with 0.1% FBS containing 2.5 μM of CERs for 48 h. Cell viability was examined using a Cell Counting Kit-8 (Dojindo, Kumamoto, Japan) according to the manufacturer’s instructions.

***Global metabolome profiling in mice***

Metabolites were extracted from plasma samples using liquid–liquid extraction procedures^25^. A total of 50 μL plasma was mixed with 375 µL of chloroform/methanol (v/v, 1/2). After centrifugation at 8000 rpm for 20 min, the upper layer of the aqueous phase and the lower layer of the organic phase were collected in a fresh tube. The solutions were dried using a vacuum centrifuge and stored at −20 ℃ until assays were performed. Dried samples were reconstituted with 50 μL mobile phase A before LC–MS/MS analysis. An LC–MS/MS system equipped with Ultimate3000 (Dionex) and LTQ-Orbitrap XL (Thermo Fischer Scientific, Waltham, MA, USA) was used to profile metabolomes in positive and negative ion modes. A reverse-phase column (Pursuit5 C18, 3 μm, 150 × 2.1 mm) was used for the analysis of organic phase solutions using mobile phase A (0.1% formic acid in H_2_O) and mobile phase B (0.1% formic acid in methanol). The LC was run at 200 µL/min and 25 °C. The separation gradient was as follows: 75% to 99.9% B for 2 min, hold at 99.9% for 7 min, 99.9% to 75% B for 0.5 min, and then hold at 75% B for 4.5 min. A HILIC column (Waters XBridge BEH amide, 2.5 μM, 2.1 × 150 mm) was used for the analysis of aqueous phase solutions using mobile phase A (10 mM ammonium acetate, 10 mM ammonium hydroxide in H_2_O/acetonitrile (v/v, 95/5), pH 9) and mobile phase B (10 mM ammonium acetate, 10 mM ammonium hydroxide in H_2_O/acetonitrile (v/v, 5/95), pH 7). Similarly, the LC was run at 200 µL/min and 25 °C. The separation gradient was as follows: hold at 30% A for 1 min, 30% to 60% A for 5 min, hold at 60% A for 6 min, 60% to 30% A for 0.1 min, and then hold at 30% A for 0.9 min.

A scan range of m/z 50–1,000 was chosen as the centroid mode, and data-derived MS/MS analysis was applied for compound annotation. Compound Discoverer software v3.3 (Thermo Fisher Scientific) extracted metabolic features and compound identification using database searches based on accurate masses with 10 ppm mass tolerance and MS/MS library matching. MetaboAnalyst 5.0 was used for pathway analysis of significantly changed metabolites (*P* < 0.05 and fold change (FC) > 1.2) in aged mice plasma after log transformation of metabolomic data.

***Targeted profiling of sphingolipids in samples from mice and cell cultures***

Muscle samples (20–30 mg) were homogenized using TissueLyzer before an internal standard solution was added. For cell lysates, ~1 million cells were harvested in 1.4 mL of cold methanol/H_2_O (4/1, v/v) after quick sequential washes with PBS and H_2_O; cells were then lysed by vigorous vortexing. Muscle tissue and plasma from mice (50 μL), as well as the CM and cell lysates of MBs, MCs, and MTs, were subjected to liquid–liquid extraction^2^ after an internal standard solution was added (50 μL of 100 nM ceramide(18:0)-d7 and sphingomyelin(18:1)-d9). Organic solutions containing sphingolipids were dried using a vacuum centrifuge and stored at –20 °C until LC–MS/MS analysis. The dried matter was reconstituted with methanol and injected into the LC–MS/MS system.

Sphingolipid metabolites were determined using an LC–MS/MS system equipped with a 1290 HPLC system (Agilent, Waldbronn, Germany) and a QTRAP 5500 (AB Sciex, Toronto, Canada). A reverse-phase column (Pursuit5 C18, 150 × 2.1 mm) was used with mobile phase A (5 mM ammonium formate/MeOH/tetrahydrofuran (5/2/3, v/v/v)) and mobile phase B (5 mM ammonium formate/MeOH/ tetrahydrofuran (1/2/7, v/v/v)). The LC was run at 200 µL/min and 35 °C; 4500 V of ion spray voltage for electrospray ionization and positive ion mode were used. The LC gradient was as follows: 50% B for 0 min, 50% B for 5 min, 50% to 70% B for 3 min, 70% B for 7 min, 70% to 90% B for 7 min, 90% B for 3 min, 90% to 50% B for 0.1 min, and 50% B for 4.9 min. Multiple reaction monitoring was performed in positive ion mode, and the extracted ion chromatogram corresponding to the specific transition for each analyte was used for quantification. The calibration range for each lipid was 0.1–10000 nM (r^2^ ≥ 0.99). Data analysis was performed using Analyst 1.5.2 software. For CM and cell lysates, lipid was normalized to total protein concentration.

***Targeted profiling of sphingolipids in human plasma***

Targeted lipidome analysis was conducted based on the hypothesis that sphingolipids in blood were candidate biomarkers for sarcopenia; 50 μL of human plasma was used, and sphingolipids were quantified as described above.

**References**

12. Murphy RA, Moore SC, Playdon M, Meirelles O, Newman AB, Milijkovic I, et al. Metabolites associated with lean mass and adiposity in older black men. *J Gerontol A Biol Sci Med Sci* 2017;**72**:1352-1359.

23. Xie WQ, He M, Yu DJ, Wu YX, Wang XH, Lv S, et al. Mouse models of sarcopenia: classification and evaluation. *J Cachexia Sarcopenia Muscle* 2021;**12**:538-554.

25. Bligh EG, Dyer WJ. A rapid method of total lipid extraction and purification. *Can J Biochem Physiol* 1959;**37**:911-917.

Supporting Information Figure legend

Supporting Information Fig. S1. Effects of ceramide (CERs) on myogenesis. (A) C2C12 myoblasts were incubated with 0 or 2.5 μM CERs for 3 days. Myotubes were stained with an anti-myosin (Skeletal, Fast) heavy chain antibody. Photographs of immunofluorescent images and quantitative results of myosin heavy chain positive area and fusion index are shown. Scale bars: 200 μm. (B) C2C12 myoblasts were incubated with 0 or 2.5 μM CERs for 2 days. Cell viability was determined using a Cell Counting Kit-8. Each bar represents the mean ± standard deviation (SD) (n = 6).

Supporting Information Table S1. Baseline characteristics of young and old mice from the aging mouse model of sarcopenia.

|  | Untargeted metabolomics | | |  | Targeted metabolomics | | |
| --- | --- | --- | --- | --- | --- | --- | --- |
|  | Young  (n = 5) | Old  (n = 5) | *P*^a^ |  | Young  (n = 10) | Old  (n = 7) | *P*^a^ |
| Age (months) | 3 | 20 |  |  | 7 | 19 |  |
| Body weight (g) | **28.06 ± 1.45** | **33.70 ± 3.84** | **0.015** |  | **30.05 ± 2.37** | **34.86 ± 2.63** | **0.001** |
| Muscle weight (g) | 0.180 ± 0.017 | 0.176 ± 0.004 | 0.674 |  | **0.197 ± 0.022** | **0.168 ± 0.042** | **0.019** |
| Relative muscle mass (%) | **0.640 ± 0.029** | **0.527 ± 0.048** | **0.002** |  | **0.663 ± 0.104** | **0.482 ± 0.050** | **0.001** |

Normally distributed data are presented as mean ± SD, with significance levels of *P* ≥ 0.05 based on the Kolmogorov–Smirnov test for normality assessment.

*P*^a^: *P*-values were obtained using Student’s t-test when normality was satisfied.

Bold numbers indicate statistically significant values.

SD, standard deviation.

Supporting Information Table S2. Metabolites identified by untargeted metabolomics in young mice and mice from the aging mouse model of sarcopenia.

Metabolic features with statistical significance (*P* < 0.05, fold change (FC) > 1.2) were included; the confidence level (CL) of compound annotation was level 2 or 3. Compound annotation was performed using library searches (HMDB, KEGG, LipidMaps, PubMed, mzCloud) based on mass accuracy (10 ppm) and MS/MS spectral matching. The ‘hp’, ‘hn’, ‘rp’, and ‘rn’ in mode represent ’hlic phase and positive ion mode’, ‘hilic phase and negative ion mode’, ‘reverse phase and positive ion mode’, and ‘reverse phase and negative ion mode’, respectively.

Supporting Information Table S3. Baseline characteristics of women in the discovery cohort (n = 72).

| Disease | Control (n = 36) | Sarcopenia (n = 36) | *P* |
| --- | --- | --- | --- |
| Age (years) | 70.4 ± 5.8 | 70.3 ± 5.7 | 0.935 |
| Weight (kg) | **65.2 [62.2; 70.1]** | **49.4 [46.0; 50.7]** | **<0.001** |
| Height (cm) | **156.0 ± 3.9** | **151.7 ± 3.8** | **<0.001** |
| BMI (kg/m^2^) | **26.8 [25.1; 29.1]** | **20.9 [19.7; 22.6]** | **<0.001** |
| Smoking, N (%) |  |  | 0.602 |
| Ex-smoker | 0 (0.0%) | 1 (2.8%) |  |
| Non-smoker | 34 (94.4%) | 35 (97.2%) |  |
| Current smoker | 1 (2.8%) | 1 (2.8%) |  |
| Drinking, N (%) |  |  | 0.170 |
| No alcohol | 7 (19.4%) | 3 (8.3%) |  |
| Alcohol < 1/week | 2 (5.6%) | 0 (0.0%) |  |
| Alcohol 1‒2/week | 0 (0.0%) | 1 (2.8%) |  |
| Alcohol ≥ 3/week | 27 (75.0%) | 32 (88.9%) |  |
| Exercise, N (%) |  |  | 0.296 |
| No exercise | 4 (11.1%) | 4 (11.1%) |  |
| Exercise < 1/week | 3 (8.3%) | 2 (5.6%) |  |
| Exercise 1‒2/week | 7 (19.4%) | 2 (5.6%) |  |
| Exercise ≥ 3/week | 22 (61.1%) | 28 (77.8%) |  |
| Hypertension, N (%) | **24 (66.7%)** | **14 (38.9%)** | **0.034** |
| Diabetes, N (%) | 29 (80.6%) | 29 (80.6%) | >0.999 |
| FM (kg) | **25.5 [22.3; 28.6]** | **16.6 [14.8; 18.6]** | **<0.001** |
| pFM (%) | **38.5 ± 5.3** | **33.3 ± 4.9** | **<0.001** |
| EQ-VAS | 6.0 [6.0; 8.0] | 6.0 [6.0; 7.0] | 0.815 |
| SARC-F | 1.0 [0.0; 2.0] | 0.0 [0.0; 2.0] | 0.545 |
| HGS (kg) | 22.2 ± 5.1 | 20.8 ± 3.8 | 0.172 |
| Chair stand up test (s) | 9.0 [7.0; 11.0] | 8.0 [6.0; 10.0] | 0.462 |
| LM (kg) | **39.1 [37.2; 41.3]** | **30.8 [29.4; 32.0]** | **<0.001** |
| ASM (kg) | **16.7 [15.8; 17.9]** | **12.3 [11.4; 13.0]** | **<0.001** |
| SMI (kg/m^2^) | **6.9 [ 6.6; 7.2]** | **5.5 [5.0; 5.5]** | **<0.001** |

Data are presented as mean ± SD for normally distributed continuous variables, median [IQR] for non-normally distributed continuous variables, or number (%) for categorical variables.

For normally and non-normally distributed continuous variables, the Student's t-test and the Mann–Whitney U test were used, respectively. Categorical variables were assessed using the chi-square or Fisher's exact test.

Non-normal distribution was confirmed by *P* < 0.05 using the Kolmogorov-Smirnov test.

Bold numbers indicate statistically significant values.

ASM, appendicular skeletal muscle mass; BMI, body mass index; EQ-VAS, EuroQol Visual Analogue Scale; FM, fat mass; HGS, hand grip strength; IQR, interquartile range; LM, lean mass; pFM, percent fat mass; SARC-F, Strength, Ambulation, Rising from a chair, stair Climbing, and history of Falling; SD, standard deviation; SMI, skeletal muscle mass index.

Supporting Information Table S4. Plasma sphingolipid levels of women in the discovery cohort based on targeted metabolome profiling (n = 72).

|  | Control (n = 36) | Case (n = 36) | log2(FC) | *P^a^* | *P^b^* |
| --- | --- | --- | --- | --- | --- |
| CER(14:0) (μM) | 14.3 ± 4.1 | 14.2 ± 3.5 | 0.010 | 0.913 | 0.913 |
| CER(16:0) (μM) | 59.8 [48.9; 73.4] | 61.5 [54.4; 74.2] | -0.071 | 0.369 | 0.990 |
| SM(16:0) (μM) | 28461.9 [24120.1; 33534.4] | 26938.3 [23576.6; 31291.5] | 0.027 | 0.691 | 0.990 |
| CER(18:0) (μM) | 39.6 ± 12.2 | 35.8 ± 11.0 | 0.145 | 0.171 | 0.870 |
| SM(18:0) (μM) | 28708.7 ± 5033.9 | 27384.3 ± 6074.9 | 0.068 | 0.317 | 0.870 |
| CER(18:1) (μM) | 8.3 ± 2.3 | 7.9 ± 2.2 | 0.075 | 0.429 | 0.870 |
| SM(18:1) (μM) | 6620.4 ± 1544.1 | 6047.1 ± 1537.9 | 0.131 | 0.119 | 0.870 |
| CER(20:0) (μM) | 99.3 [80.6; 114.8] | 92.4 [78.5; 115.8] | 0.028 | 0.780 | 0.990 |
| CER(24:0) (μM) | 2047.5 ± 526.7 | 1981.4 ± 550.7 | 0.047 | 0.604 | 0.870 |
| SM(24:0) (μM) | 15259.3 [12205.1; 17778.9] | 14406.0 [12505.6; 17425.2] | 0.024 | 0.788 | 0.990 |
| CER(24:1) (μM) | 645.9 ± 207.7 | 625.9 ± 149.6 | 0.045 | 0.641 | 0.870 |
| SM(24:1) (μM) | 32695.1 [29379.9; 41872.2] | 34471.3 [31648.5; 39260.1] | 0.018 | 0.992 | 0.992 |

Data are presented as mean ± SD for sphingolipid levels with normal distribution and as median [IQR] for sphingolipid levels with non-normal distribution, with significance levels of *P* < 0.05 based on the Kolmogorov–Smirnov test for normality assessment.

*P*^a^: The *P*-value was calculated using Student's t-test and Mann–Whitney U test for normally and non-normally distributed continuous variables, respectively.

*P*^b^: *P*-value after adjusting for multiple testing corrections using the false discovery rate (FDR) method.

CER, ceramide; FC, fold change; IQR, interquartile range; SD, standard deviation; SM, sphingomyelin.

Bold numbers indicate statistically significant values.

Supporting Information Table S5. Plasma dihydroceramide levels and amino acid (alanine, serine, glycine) and deoxyceramide levels in men in the discovery cohort based on targeted metabolome profiling (n = 144).

|  | Control (n = 72) | Case (n = 72) | log2(FC) | *P^a^* | *P^b^* |
| --- | --- | --- | --- | --- | --- |
| Canonical pathway |  |  |  |  |  |
| DihydroCER(14:0) | 56.2 [39.1;84.8] | 58.8 [40.8;128.6] | -0.317 | 0.307 | 0.604 |
| DihydroCER(16:0) | 36.5 [24.8;49.5] | 38.3 [29.0;60.1] | -0.210 | 0.306 | 0.604 |
| DihydroCER(18:0) | 106.0 [61.3;241.8] | 113.3 [58.4;216.8] | -0.155 | 0.930 | 0.930 |
| DihydroCER(18:1) | 3.7 [ 2.7; 5.5] | 3.9 [ 3.0; 5.3] | -0.105 | 0.402 | 0.604 |
| DihydroCER(24:0) | 2691.1 [1887.9;6401.2] | 3368.1 [1850.0;6834.9] | -0.201 | 0.774 | 0.928 |
| DihydroCER(24:1) | 475.9 [285.5;1233.3] | 656.1 [347.1;1068.6] | -0.335 | 0.373 | 0.604 |
| Non-canonical pathway |  |  |  |  |  |
| Alanine (μM) | 837.5 [720.0;1029.9] | 793.6 [659.6;1012.9] | 0.037 | 0.359 | 0.646 |
| Serine (μM) | **106.6 [98.8;125.4]** | **119.1 [98.4;142.8]** | -0.119 | **0.048** | 0.387 |
| Glycine (μM) | 96.1 [84.5;106.2] | 92.8 [77.7;111.1] | -0.004 | 0.698 | 0.727 |
| DeoxyCER(16:0) | 19.6 [13.0;23.8] | 17.7 [12.7;24.9] | 0.057 | 0.484 | 0.646 |
| DeoxyCER(24:0) | 7.0 [ 4.2;13.2] | 7.9 [ 3.8;21.2] | -0.719 | 0.256 | 0.646 |
| DihrodeoxyCER(24:0) | 256.1 [149.3;416.9] | 285.2 [130.7;716.8] | -0.537 | 0.462 | 0.646 |
| DeoxyCER(24:1) | 391.9 [200.3;575.1] | 353.7 [199.8;812.2] | -0.456 | 0.727 | 0.727 |
| DihrodeoxyCER(24:1) | 125.5 [70.3;200.2] | 129.4 [68.2;314.4] | -0.682 | 0.326 | 0.646 |

Data are presented as median [IQR] for sphingolipid levels with non-normal distributions, with significance levels of *P* < 0.05 based on the Kolmogorov–Smirnov test for normality assessment.

*P*^a^: *P*-value from the Mann–Whitney U test for non-normally distributed continuous variables.

*P*^b^: *P*-value after adjusting for multiple testing corrections using the false discovery rate (FDR) method.

CER, ceramide; FC, fold change; IQR, interquartile range; SD, standard deviation.

Bold numbers indicate statistically significant values.

Supporting Information Table S6. Baseline characteristics of men in the validation cohort (n = 164).

| Disease | Control (n = 128) | Sarcopenia (n = 36) | *P* |
| --- | --- | --- | --- |
| Age (years) | 63.0 [60.0; 68.0] | 63.5 [60.0; 69.5] | 0.714 |
| Weight (kg) | **70.9 [64.6; 75.3]** | **59.0 [54.2; 62.5]** | **<0.001** |
| Height (cm) | 168.7 ± 5.7 | 167.2 ± 5.3 | 0.159 |
| BMI (kg/m^2^) | **24.4 [23.3; 26.1]** | **20.6 [19.4; 22.7]** | **<0.001** |
| Smoking, N (%) |  |  | 0.266 |
| Ex-smoker | 89 (69.5%) | 24 (66.7%) |  |
| Non-smoker | 23 (18.0%) | 4 (11.1%) |  |
| Current smoker | 16 (12.5%) | 8 (22.2%) |  |
| Drinking, N (%) |  |  | 0.154 |
| Alcohol < 3 units/day | 85 (66.4%) | 29 (80.6%) |  |
| Alcohol ≥ 3 units/day | 43 (33.6%) | 7 (19.4%) |  |
| Exercise, N (%) |  |  | 0.575 |
| < 30 mins/day or < 3/week | 48 (37.5%) | 16 (44.4%) |  |
| ≥ 30 mind/day and ≥ 3/week | 80 (62.5%) | 20 (55.6%) |  |
| Hypertension, N (%) | 67 (52.3%) | 15 (41.7%) | 0.346 |
| Diabetes, N (%) | 19 (14.8%) | 5 (13.9%) | >0.999 |
| FM (kg) | **16.5 [13.8; 20.4]** | **12.8 [9.7; 15.5]** | **<0.001** |
| pFM (%) | 23.9 ± 5.4 | 22.1 ± 6.6 | 0.084 |
| Measurement of HGS, N (%) | **50 (39.1%)** | **24 (66.7%)** | **0.003** |
| HGS (kg) | **35.2 [31.7; 39.9]** | **32.1 [30.1; 34.6]** | **0.013** |
| LM (kg) | **50.2 [46.8; 53.8]** | **42.9 [41.1; 44.5]** | **<0.001** |
| ASM (kg) | **22.4 [20.8; 24.3]** | **18.9 [18.2; 20.4]** | **<0.001** |
| SMI (kg/m^2^) | **7.9 [7.5; 8.3]** | **6.9 [6.8; 7.0]** | **<0.001** |
| SM(16:0) (μM) | **47238.7 [42355.7; 53508.9]** | **58733.2 [48464.8; 74411.6]** | **<0.001** |
| CER(24:1) (μM) | **734.9 [643.8; 866.5]** | **853.9 [660.8; 975.2]** | **0.029** |
| SM(24:1) (μM) | **37471.3 [30170.9; 42998.4]** | **44511.7 [35729.5; 55065.6]** | **0.001** |

Data are presented as mean ± SD for normally distributed continuous variables, median [IQR] for non-normally distributed continuous variables with significance levels of *P* < 0.05 based on the Kolmogorov–Smirnov test for normality assessment, or number (%) for categorical variables.

We used the Student's t-test for continuous variables when normality was satisfied and the Mann–Whitney U test for a non-normal distribution. For categorical variables, we used the chi-square or Fisher's exact test.

ASM, appendicular skeletal muscle mass; BMI, body mass index; EQ-VAS, EuroQol Visual Analogue Scale; FM, fat mass; HGS, hand grip strength; IQR, interquartile range; LM, lean mass; pFM, percent fat mass; SARC-F, Strength, Ambulation, Rising from a chair, stair Climbing, and history of Falling; SD, standard deviation; SMI, skeletal muscle mass index.

Bold numbers indicate statistically significant values.
